# Microbiota Emergencies in the Diagnosis of Lung Diseases: A Meta-Analysis

**DOI:** 10.3389/fcimb.2021.709634

**Published:** 2021-09-21

**Authors:** Renyu Ruan, Xiangmin Deng, Xiaoyan Dong, Qi Wang, Xiaoling Lv, Caijuan Si

**Affiliations:** ^1^College of Undergradute, Jiangsu Food & Pharmaceutical Science College, Huaian, China; ^2^College of Pharmacy and Traditional Chinese Medicine, Jiangsu College of Nursing, Huaian, China; ^3^College of Pharmacy, Harbin Medical University–Daqing, Da Qing, China; ^4^Department of Nutrition, Zhejiang Hospital, Hangzhou, China

**Keywords:** lung microbiota, COPD, asthma, diagnosis, meta-analysis

## Abstract

Although many studies have reported that microbiota emergencies are deeply involved in the occurrence and subsequent progression of lung diseases, the present diagnosis of lung disease depends on microbiota markers, which is still poorly understood. Therefore, a meta-analysis was performed to confirm lung microbiota markers for the diagnosis of lung diseases. Literature databases were searched following the inclusion and exclusion criteria. There are 6 studies including 1347 patients and 26 comparisons to be enrolled, and then the diagnostic effect was evaluated using Stata 14.0 and Meta-disc 1.4 software. The pooled sensitivity (SEN), specificity (SPE), diagnostic likelihood ratio positive (DLR+), diagnostic likelihood ratio negative (DLR−), and diagnostic OR (DOR), as well as area under the curve (AUC) of microbiota markers in the diagnosis of lung diseases were 0.90 (95% CI: 0.83-0.94), 0.89 (95% CI: 0.76-0.95), 7.86 (95% CI: 3.39-18.21), 0.12 (95% CI: 0.06-0.21), 22.254 (95% CI: 12.83-39.59.14), and 0.95 (95% CI: 0.93-0.97), respectively. Subgroup analysis revealed that research based on Caucasian, adult, BAL fluid, PCR, pneumonia obtained higher AUC values. The microbiota markers have shown potential diagnosis value for lung diseases. But further large-scale clinical studies are still needed to verify and replicate the diagnostic value of lung microbiota markers.

## Introduction

The human microbiota exists both inside and outside the human body, which is an ecosystem of microorganisms consisting of bacteria, archaea, and eukarya (Wolff et al., 2018). The bacterial microbiota had been widely studied because of its multiple of functions. In the meantime, the microbiome, the genetic content of microbiota, also had been widely investigated following the development of next-generation sequencing (Wolff et al., 2018). Microbiota composition often plays a key emerging factor to affect the immune responses of patients as well as healthy individuals. However, the lung microbiota had been less noticed compared with other organs in that healthy lungs were long thought to be sterile. And now, more and more studies reported that there existed multiple kinds of bacteria in healthy lungs (Huffnagle et al., 2017). Dysbiosis of respiratory system microbiota can lead to the occurrence or deterioration of lung diseases (Liu et al., 2021). In detail, the local immune system could be affected by the dysbiosis of lung microbiota, which determines the balance of inflammation (Kramer & Genco, 2017). Moreover, different microorganisms cause different host immune responses. However, the microbiome not only acts on the organism by immunity responses, inflammation response, or metabolism mechanism, but also on the colonized organs locally (Ran et al., 2020). For instance, fungal colonization and bacterial flora colonization were interacted with each other, which promoted the mycelial growth of *fungi* through colonized bacteria secreting antifungal substances and easily causing impaired lung infection (Oever & Netea, 2014). Moreover, the colonized bacteria reduced the release of the macrophages and the aggregation of neutrophils, which in turn affected the colonization of *fungi*. For example, *A. fumigates* has been found to be related to the impaired lung function in severe asthmatics (Agbetile et al., 2012; Man et al., 2019). In addition, impairment in host immune systems in immunocompetent and immunocompromised patients, including those with chronic obstructive pulmonary disease (COPD) and asthma, also increased the susceptibility to fungal infections (Man et al., 2019).

16S rRNA gene sequencing was the first application tool in the Human Microbiome Project (HMP) initiated in 2007 by the U.S. National Institutes of Health. The insight gained thus far into the lung microbiome in diseased states has led to considerable interest in the potential development of biomarkers, improved diagnostics, and therapies (Moffatt & Cookson, 2017). However, 16S rRNA did not provide closely related species, such as streptococcal species. Therefore, a recent study provided some species-level data by using quantitative PCR to confirm the potentially pathogenic OTUs (Man et al., 2019). A majority of preliminary investigations studied the relationship of bacteria in the respiratory system and lung diseases as well as its potential diagnostic value. For example, in increasing abundance of *Moraxella* and *Haemophilus influenza* directly reduced the occurrence and exacerbation of COPD (Ahearn et al., 2017; Mayhew et al., 2018), which showed their potential clinical diagnostic value. Jinho Yang et al. also found that *S. aureus*, *A. baumannii*, *E. cloacae*, and *P. aeruginosa* predominantly affected the progress of COPD and asthma diseases, which were the most abundant organisms at the genetic expression level. In addition, there were diagnostic values of 0.73 and 0.78 of AUC in asthma and COPD, independently (Yang et al., 2020). The microbial community of the human respiratory tract can exist in the mouth, the nose, the upper respiratory tract, and the lungs. Pulmonary microenvironment often changes following the breathing movement, which causes the lungs to have direct communication with the outside environment, thus, in turn, the microbial community of the whole respiratory system was also changed (Budden et al., 2019). Previous investigation found that lower respiratory tract infection caused an acute exacerbation in 82% of COPD patients (Choi et al., 2013). The infections’ progress was approximately caused by bacteria such as *Haemophilusinfluenzae*, *Moraxella catarrhalis*, *Streptococcus pneumoniae*, *Pseudomonasaeruginosa*, and so on. Meanwhile, a virus caused aggravation of the lung disease, such as primarily human *Rhinovirus* (HRV), *Influenza virus*, *Coronavirus*, and *respiratory syncytial virus* (RSV) (Bouquet et al., 2020). Also many investigations reported that bacteria caused secondary bacterial lung infection (Lu et al., 2017), which included *Haemolyticus, Streptococcus pneumoniae, nonliquefaciens, Haemophilus, Filifactor, Megasphaera*, and so on. All of these showed a significant difference in abundance between the H7N9 and HC groups, with diagnostic AUC values of >0.70.

Traditionally, diagnostics for the detection of potentially pathogenic viruses and bacteria cover only some pathobionts and discriminate poorly between patients and healthy subjects, or are time consuming, for example, pneumonia and respiratory tract infections. If a microbiota-based diagnostic or classification tool could improve accuracy and timely diagnosis for diseases, it would have major implications for clinical treatment. There are numerous studies on microbiota biomarkers related to lung diseases, and the diagnostic value of potential microbial markers in lung diseases has also been analyzed (Agbetile et al., 2012; Bafadhel et al., 2014; May et al., 2015; Lu et al., 2017; Man et al., 2019; Yang et al., 2020). However, the small sample size in the included studies limited the interpretation of diagnostic value of these microbiota biomarkers. In order to systematically describe the potential diagnostic value of microbial markers in lung diseases, we systematically searched the literature database and performed the present meta-analysis. Studies were included following the inclusion and exclusion criteria. Then we extracted data from these studies. Diagnostic indicators including SEN, SPE, DLR+, DLR-, and AUC were calculated, which followed by sensitivity analyses and publication bias assessment.

## Materials and Methods

The present meta-analysis was guided by the Preferred Reporting Items for Systematic Reviews and Meta-Analysis (PRISMA) guidelines (Moher et al., 2009).

### Search Strategy

Two authors independently conducted the literature search in PubMed, Web of Science, EMbase, PMC, Google Scholar, Cochrane Library, the Chinese National Knowledge Infrastructure (CNKI), and the Chinese Biomedical Literature Database (CBM). The literature was limited to studies in English or Chinese published before March 3, 2021. The following key terms were used for the search: “lung microbiota”, “lung microbiome”, “respiratory microbiome”, “bacteria”, “COPD”, “asthma”, “respiratory infection diseases”, “diagnosis”, “diagnostic value”, “sensitivity”, “specificity”, “AUC”, and “ROC”. Articles in the references were also searched to avoid missing the relevant studies.

### Inclusion and Exclusion Criteria

Searched articles were included if they met the following criteria: (1) included all types of respiratory infectious diseases, or COPD, or asthma; (2) the diagnosis of these diseases was clinically confirmed according to the diseases guidelines; (3) the study included healthy individuals as a control group; (4) lung microbiota or lung microbiome for the diagnosis of lung diseases were evaluated; (5) case group size, control group size, and sensitivity and specificity were provided; (6) the language was limited to Chinese or English. Studies were excluded with the following characteristics: (1) case reports, comments, review articles; (2) repeated studies; (3) insufficient data to calculate the sensitivity and specificity; and (4) studies not related to the topics.

### Data Extraction and Quality Assessment

The following data was retrieved by two authors from the included studies: first author, publication year, country, sample characteristic, sample source, detection content and microbiome detection method, the case and control size, and the true-positive (TP), false-positive (FP), true-negative (TN), and false-negative (FN) value. If the included studies did not provide detailed data, TP, FP, TN, and FN would be calculated based on the sensitivity, specificity, and the sample’s size. If there was any inconsistency, the third researcher checked and resolved.

The Quality Assessment of Diagnostic Accuracy Studies 2 (QUADAS-2) tool was used to assess the quality of the included studies in RevMan 5.3 software (Whiting et al., 2011). The QUADAS-2 included patient selection, index test, reference standard, and flow and timing, including 14 questions about the risk of bias of the included article. The quality of the literature was also independently assessed by two authors.

### Statistical Analysis

The present meta-analysis was conducted by using Stata 12.0 (Stata Corporation, College Station, TX), RevMan 5.3 (https://community.cochrane.org/help/tools-and-software/revman-5) and Meta-DiSc 1.4 (XI Cochrane Colloquium, Barcelona, Spain) software. Diagnostic indicators, including the pooled SEN, SPE, and its related DLR+, DLR-, and DOR were calculated using a random effect model (Reitsma et al., 2005). The summary receiver operator characteristic (SROCs) curves and the pooled area under the curve (AUC) values with the related corresponding 95% confidence intervals (CIs) were also calculated (Hamza et al., 2009). In addition, the Higgin’s I^2^ and Cochran’s Q tests were also performed during this analysis (Higgins et al., 2003). Deeks’ funnel plot was used to determine publication bias. Fagan’s nomogram was generated to evaluate the post-test probability. It was considered significantly different when *P* value was <0.05.

## Results

### Characteristics and Quality of the Included Studies

**Figure 1:**
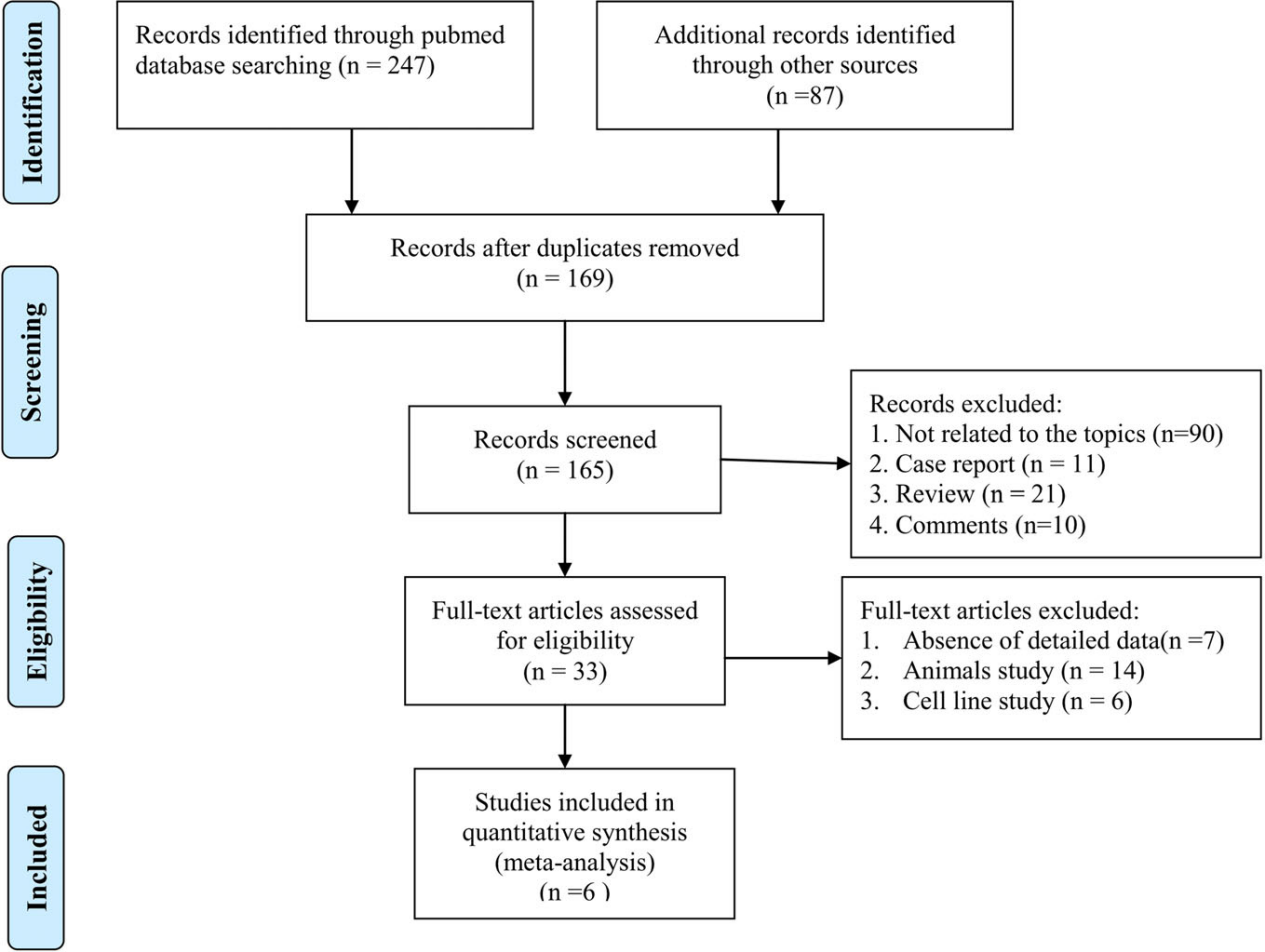
Flowchart of studies for selecting process from databases.

After the literature search, 334 articles were obtained from the online databases. Then, 169 articles were removed because of duplication. Then, 132 articles were excluded because they did not meet the inclusion criteria. After reading the full text, we removed another 27 articles that lacked detailed data to calculate TP, FP, TN, or FN values. Finally, 6 articles were included in this meta-analysis. The detailed literature selection procedure is shown in **Figure 1**.

The characteristics of the patients and health subjects as well as the included studies are shown in **Table 1**. This meta-analysis collected 1347 individuals (873 patients and 474 healthy individuals) involving a total of 26 comparisons. The publication year of the included studies ranged from 2012 to 2020, and were conducted in U.K., U.S., Korea, Netherlands, and China. The lung microbiome sources included nasopharyngeal swab, oropharyngeal swab, sputum, BAL fluid, and serum samples. DNA was extracted from the microbiome and quantitative reverse transcription polymerase chain reaction (qRT-PCR) was used to detect the content of each microbiome. The number of case and control group of each study ranged from 10 to 306, and the respiratory diseases included ventilator-associated pneumonia, asthma, COPD, lower respiratory tract infections, and secondary bacterial lung infection. In addition, Addison et al. showed seven kinds of bacteria in lung microbiota (May et al., 2015). Lu et al., (2017) showed 13 kinds of bacteria in lung microbiota. Yang et al. and others reported five kinds of bacteria (*A. fumigatus, S. aureus, A. baumannii, E. cloacae, P. aeruginosa*) in the diagnosis of COPD and asthma (Agbetile et al., 2012; Yang et al., 2020). Man et al. found that *Haemolyticus*, *Streptococcus pneumoniae*, and *Nonliquefaciens* showed potential diagnostic value in lower respiratory tract infections (Man et al., 2019).

**Table 1:** Characteristics of the included studies.

First author	Year	Country	Study design	Disease	Study group	No.	Age of cases	Other infection in cases	Sample source	Microbiota type	Detection content	Detection method	QUADAS-2 score
Addison K. May	2014	U.S.	Pilot study	VAP	Ventilator-associated pneumonia patients	48	Adult	No	BAL fluid	*Acinetobacterbaumannii*, *Escherichia coli*, *Enterococcus faecalis*, *Enterococcus faecium*, *Klebsiella pneumonia*, *Pseudomonas aeruginosa*, *1.1 Staphylococcus aureus*	DNA	PCR	5
					Mechanically ventilated patients	48							
					HC	10							
Jinho Yang	2020	Korea	Case-control study	Asthma COPD	Asthma	239	55.5 ± 14.5	No	Serum	*S. aureus*, *A. baumannii*, *E. cloacae*, *P. aeruginosa*	Bacterial EV immune- globulin G	16S rRNA gene ELISA	3
					COPD	205	66.4 ± 7						
					HC	88	50.8 ± 9.8						
Wing Ho Man	2019	Netherlands	Prospective study	LRTIs	LRTIs	151	4.9-27.4 M	No	Nasopharyngeal	*haemolyticus*, *Streptococcus* *pneumoniae, nonliquefaciens*	DNA	16S rRNA gene	5
					HC	306	5.3-28.4 M						
Haifeng Lu	2017	China	Case-control study	SBLI	H7N9 patients with SBLI	21	60.5 ± 13.5	Co-infection with flavobacterium indologenes, staphylococcus Candida albicans	Oropharyngeal swab	*Haemophilus* *Filifactor*, *Megasphaera*, *Leptotrichia* *tannerella* *bacteroides* *Leptotrichia* *Oribacterium* *Streptococcus* *Atopobium* *Eubacterium* *Haemophilus* *Solobacterium* *1.2 Rothia*	DNA	16S rRNA gene	2
					H7N9 patients without SBLI	30	53 ± 12.7						
					HC	30	50 ± 9.3						
J. Agbetile	2012	U.K.	Case-control study	Asthma	Asthma	68	58 (24–83)	No	Sputum	*A. fumigatus*	DNA	PCR	4
					HC	18	40 (21–67)						
Mona Bafadhel	2017	U.K.	Case-control study	COPD	COPD	63	72 (53–86)	No	Sputum	*A. fumigatus*	DNA	PCR	4
					HC	22	58 (41–79)						

VAP, ventilator-associated pneumonia; LRTIs, lower respiratory tract infections; SBLI, secondary bacterial lung infection; BAL, broncho-alveolar lavage; HC, healthy control; COPD, chronic obstructive pulmonary disease.

As shown in **Supplementary Figure 1**, the results of the QUADAS-2 study quality assessment indicated that the quality of the included studies was convincing. The quality scores by the QUADAS-2 tool are summarized in **Table 1**. There are four included studies with a score ≥ 4, and two included studies with a score ≤ 4. Therefore, we included two poor quality studies and four better quality studies.

### Diagnostic Performance

**Figure 2:**
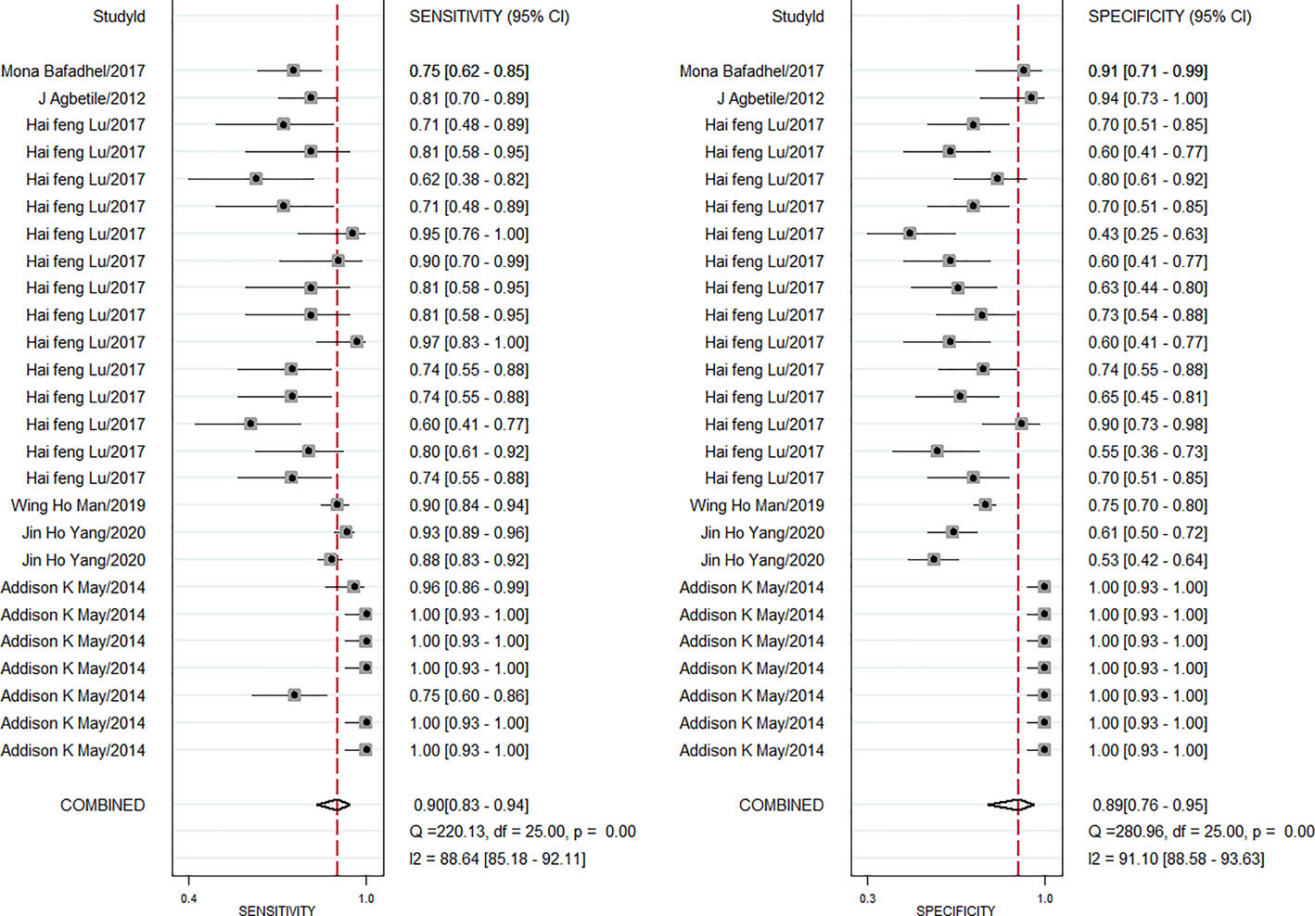
Forest plot of sensitivity and specificity of microbiota biomarkers for the diagnosis of lung diseases.

**Figure 3:**
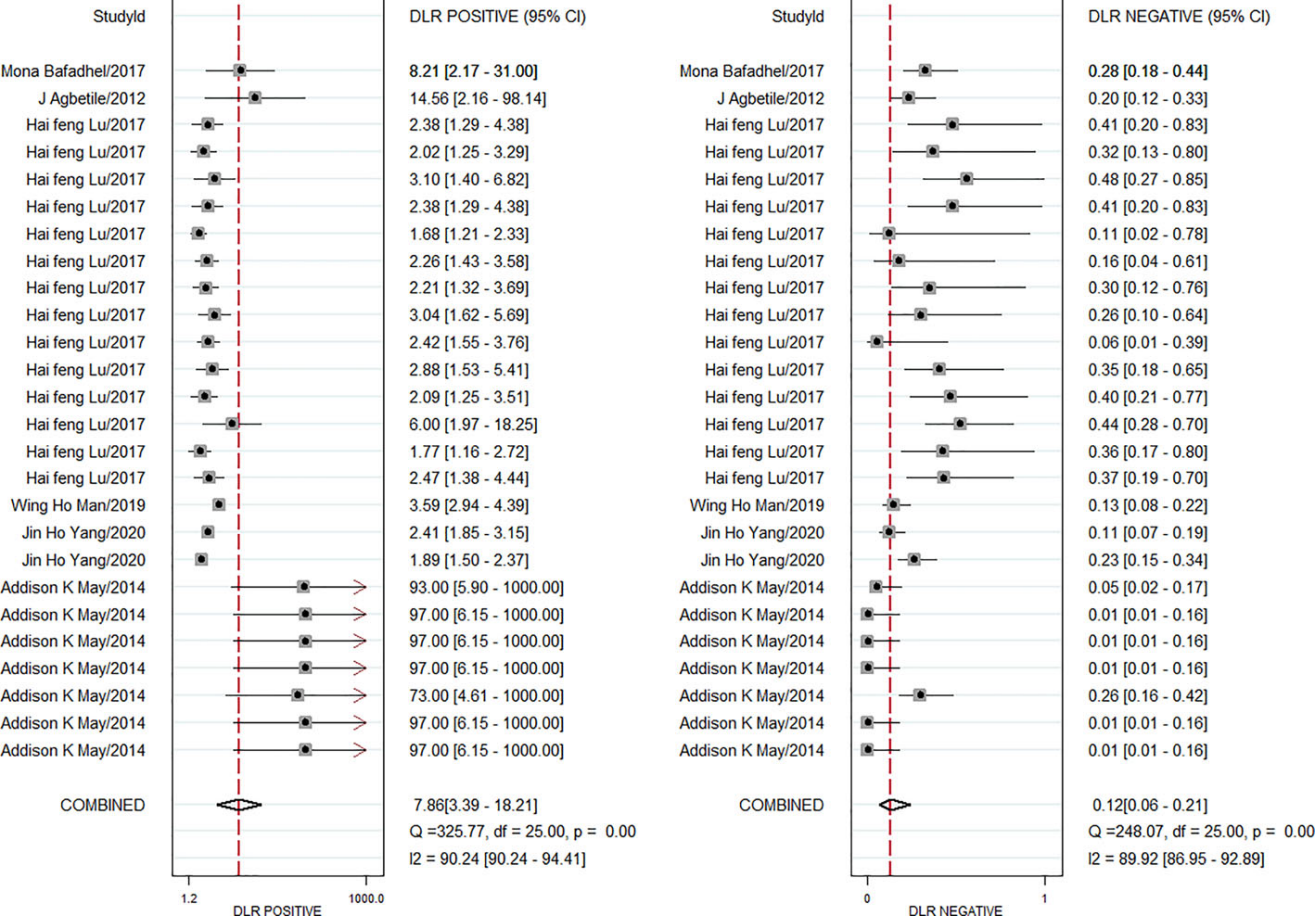
Forest plot of DLR^+^ and DLR^−^ of microbiota biomarkers for the diagnosis of lung disease.

**Figure 4:**
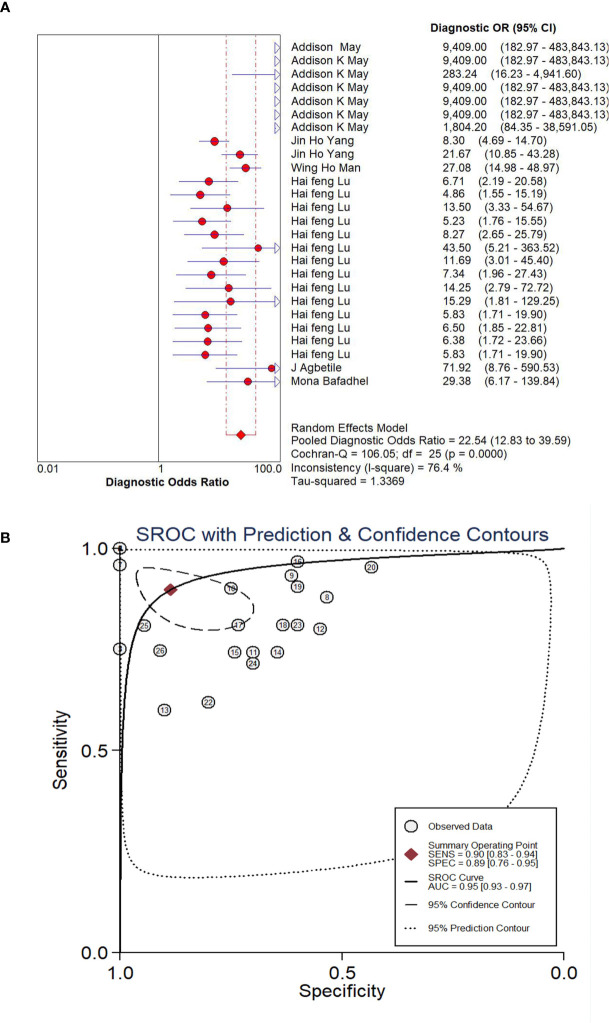
Forest plot of DOR **(A)** and SROC **(B)** of microbiota biomarkers for the diagnosis of lung disease.

The pooled diagnostic effect of these 26 comparisons was evaluated by a random effects model. The values of the pooled SEN and SPE are shown in **Figure 2**. The pooled SEN was 0.90 (95% CI: 0.83-0.94, *I^2^ = *88.64%, P<0.05) and the pooled SPE was 0.89 (95% CI: 0.76-0.95, *I^2^ = *91.10%, P<0.05). **Figure 3** illustrates the values of the pooled DLR+ and DLR-. The pooled DLR+ was 7.86 (95% CI: 3.39-18.21, *I^2^ = *90.24%, P<0.05) and the pooled DLR- was 0.12 (95% CI: 0.06-0.21, *I^2^ = *89.92%, P<0.05). The DOR value was 22.54 (95% CI: 12.83-39.59, *I^2^ = *76.4%, P<0.05; **Figure 4A**) and the AUC was 0.95 (95% CI: 0.93-0.97; **Figure 4B**). These findings described that lung microbiota biomarkers played an important role in diagnosis of lung diseases. But the Cochran’s Q values suggested significant heterogeneity during the analysis.

### The Assessment of Heterogeneity

The heterogeneity analysis across studies was used for the Cochran Q and I^2^ tests. Results showed that the Cochran Q was 69.923 (p < 0.05) and I^2^ value was 97 (95% CI, 95–99). The Cochran Q and I^2^ values of sensitivity were 220.13 (p < 0.05) and 88.64 (95% CI, 85.18-92.11), respectively. The Cochran Q and I^2^ values of specificity were 280.96 (p < 0.05) and 91.10 (95% CI, 88.58–93.63) (**Figure 2**), respectively. Therefore, the results suggested that there was significant heterogeneity between the included studies. Then, subgroup analysis was conducted to explore the potential heterogeneity. The ethnicity (Caucasian or Asian), age of case subjects (children, adult, and old population), detection method (PCR or ELISA), sample source (BAL fluid, serum, mouth), type of lung disease (pneumonia, lung infection, COPD, asthma) were used as covariates to perform the subgroup analysis using Meta-disc 1.4 software. As shown in **Table 2**, in the subgroup based on ethnicity, lung microbiota markers had higher diagnosis ability for Caucasian than for Asian populations, which was with higher sensitivity (0.91 *vs*. 0.86), specificity (0.99 *vs*. 0.68), and AUC (0.99 *vs*. 0.81) in the Caucasian population. In the analysis of lung microbiota source, microbiota isolated from BAL fluid seemed to be the optimal source because of its high sensitivity, specificity, and AUC, which were 0.96, 0.99, and 0.99, respectively. However, we also obtained a significant diagnostic value from the mouth microbiota, which was 0.81 of AUC. For the subgroup based on the detection method of lung microbiota, we found differences between PCR and ELISA detection. Sensitivity (0.86), specificity (0.80), and AUC (0.93) were shown with good diagnostic values in the PCR method. Moreover, the distinctions between different lung diseases in microbiota markers extracted from six studies were also performed. The comparison showed a series of good sensitivity (0.96, 0.81, 0.89, and 0.86) for pneumonia, lung infections, COPD, and asthma, but a poor specificity (0.70, 0.67, 0.60) for lung infections, COPD, and asthma. However, there were only two comparisons during subgroup analysis, and we could not obtain an effective AUC value, which may also limit the interpretation of our results.

**Table 2:** Results of subgroup analysis in diagnostic meta-analysis.

Subgroups	No. comparisions	SEN (95% CI)	SPE (95% CI)	DLR+ (95% CI)	DLR- (95% CI)	AUC
Ethnicity						
Caucasian	9	0.91 (0.88 -0.93)	0.99 (0.98-0.99)	37.53 (15.90-88.58)	0.06 (0.02-0.17)	0.99
Asian	17	0.86 (0.83-0.88)	0. 68 (0.65-0.71)	2.36 (2.04-2.74)	0.28 (0.21-0.37)	0.81
Age						
Adult	17	0.89 (0.86-0.91)	0.82 (0.78-0.84)	5.26 (2.97-9.33)	0.17 (0.10-0.28)	0.96
Old	8	0.84 (0.80-0.87)	0.68 (0.63-0.74)	2.44 (1.97-3.01)	0.29 (0.19-0.43)	0.83
Sample type						
BAL fluid	7	0.96 (0.93-0.98)	0.99 (0.98-0.99)	92.59 (32.64-262.65)	0.02 (0.00-0.16)	0.99
Serum	2	0.90 (0.87-0.93)	0.57 (0.50-0.65)	2.11 (1.66-2.69)	0.16 (0.08-0.33)	-
Mouth	16	0.78 (0.74-0.81)	0.69 (0.64-0.730	2.36 (1.98-2.81)	0.33 (0.28-0.39)	0.81
Method						
PCR	24	0.86 (0.84-0.88)	0.80 (0.78-0.82)	3.83 (2.67-5.51)	0.21 (0.15-0.31)	0.93
ELISA	2	0.90 (0.87-0.93)	0.57 (0.50-0.65)	2.11 (1.66-2.69)	0.16 (0.08-0.33)	-
Diseases						
Pneumonia	7	0.96 (0.93-0.98)	0.99 (0.98-0.99)	92.59 (32.64-262.65)	0.02 (0.00-0.16)	0.99
Lung infection	15	0.81 (0.18-0.85)	0.70 (0.67-0.73)	2.43 (2.05-2.89)	0.31 (0.26-0.41)	0.81
COPD	2	0.89 (0.84-0.92)	0.67 (0.58-0.76)	3.79 (1.11-12.99)	0.18 (0.07-0.47)	-
Asthma	2	0.86 (0.93-0.98)	0.60 (0.98-0.99)	4.39 (0. 47-41.33)	0.22 (0.26-0.30)	-

### Clinical Diagnostic Value of Lung Microbiota Biomarkers in Lung Diseases

**Figure 5:**
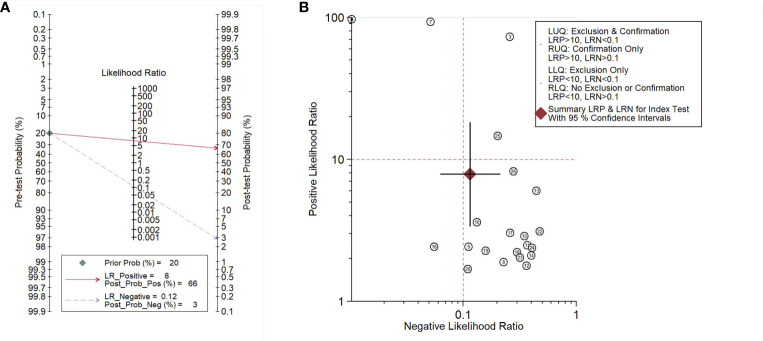
Fagan’s nomogram assessing **(A)** and dot plot of negative likelihood ratio **(B)** of microbiota biomarkers for the diagnosis of lung disease.

To illustrate the diagnostic value of lung microbiota markers in lung diseases, we performed the Fagan nomogram analysis. As shown in **Figure 5A**, the prior probability, likelihood ratio, and post-probability were 20%, 8, and 66%, respectively. In addition, shown as **Figure 5B**, when the upper left limit LRP was >10 and the LRN < 0.1 in the likelihood ratio dot plot, it was confirmed and excluded, for example, the comparison of 7. When LRP was > 10 and the LRN was also > 0.1, it was confirmed only, for example, the comparison of 3 and 25. When LRP was < 10 and the LRN was also <0.1, it was excluded only, for example, the comparison of 16. When LRP was < 10 and the LRN was also > 0.1, there was no exclusion and confirmation. In conclusion, it is reliable basically and the microbiota could play the potential biomarkers in the diagnosis of lung diseases.

### Sensitivity Analysis and Publication Bias

**Figure 6:**
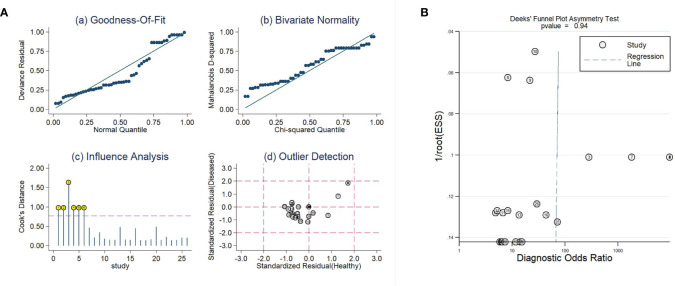
The sensitivity analysis **(A)** and Deeks’ funnel plot asymmetry test **(B)** of this meta-analysis.

As shown in **Figure 6A**, influence analysis indicated that six comparisons were out of the limit. However, after removing the six comparisons, the diagnostic parameters were changed but comparable (sensitivity: 0.83 *vs*. 0.90; specificity: 0.72 *vs*. 0.89; AUC: 0.86 *vs*. 0.95). Therefore, the analysis results were basically reliable. An analysis of publication bias was also performed. As shown in **Figure 6B**, the *P* value was 0.94 in Deek’s funnel plot, which suggested that there was no publication bias.

## Discussion

There have always been controversies whether potential microbiota biomarkers could be applied in several lung diseases. Therefore, we present an integrative meta-analysis to illustrate the diagnostic role of potential microbiota biomarkers in COPD and asthma as well as lung infection diseases. Six eligible articles including 26 comparisons were enrolled in the present study. The pooled sensitivity in six studies was 0.90 (95% CI, 0.83-0.94) and the pooled specificity was 0.89 (95% CI, 0.76-0.95). The pooled DLR+ in six studies was 7.86 (95% CI, 3.39-18.21) and the pooled DLR- was 0.12 (95% CI, 0.06-0.21). The AUC was 0.95 (95% CI, 0.93-0.97). Our findings indicate that microbiota biomarkers can be novel biological molecules in the diagnosis of lung diseases. According to the results of this analysis, the reproducibility of the *Acinetobacter baumannii*, *Escherichia coli*, *Enterococcus faecalis*, and so on, in segregating lung diseases patients *vs*. controls implicated the possibility of microbiota markers for lung diseases diagnostics.

There are multiple kinds of diagnostic biomarkers clinically used in lung diseases, for example, circulating tumor DNA (ctDNA), circulating tumor cells, microRNA, and lncRNAs. Previous reports illustrated that patients with lung cancer who had detectable ctDNA presented a higher risk of recurrence (Lee et al., 2019). Lee et al. also showed that miR-1248 played an up regulator to increase the expression of IL-5 in asthma patients and was a potential useful diagnostic indicator for asthma disease (Panganiban et al., 2012). Another previous study suggested that IL-8 was highly sensitive and vascular endothelial growth factor (VEGF) was highly specific, which had been used in the diagnosis of asthma-COPD overlap syndrome (Ding et al., 2020). Thymic stromal lymphopoietin (TSLP), thymus and activated chemokine (TARC), and IL-8 had also been used in the diagnosis of antifungal treatment of patients with asthma-induced ABPA (Kozlova et al., 2020). In addition, extracellular vesicles (EVs), one kind of novel intercellular transporter, had also been confirmed in the role of diagnostic value in COPD, cystic fibrosis (CF), asthma, and lung cancer (Trappe et al., 2021).

Compared with traditional biomarkers such as IL-5, TSLP, and TARC, microbiota biomarkers also represent a vital role in the development and progress of lung diseases. For instance, *Mycobacterium* species combined with a specific bacterial community contributed the onset, progression, recurrence, and outcome of pulmonary tuberculosis. *R. mucilaginosa*, the predominant bacterial community of the upper respiratory tract, led the occurrence of pneumonia and bacteremia in patients that were immunocompromised (Maraki & Papadakis, 2015). These bacteria had also been found to have a diagnostic role in pulmonary tuberculosis (Hong et al., 2018). In addition, *Veillonella*, *Megasphaera*, and *Streptococcus* presented a remarkably higher content in lung cancer patients than in healthy individuals. Similarly, *Neisseria*, *Staphylococcus*, and *Dialister* showed a higher level in lung cancerous lesions than in normal lung tissues (Ran et al., 2020). However, different from the present study, one report found that *Proteobacteria*, *Actinobacteria*, and *Firmicutes* predominantly promoted the development of COPD by contributing the biosynthesis of palmitate, homocysteine, and urate (Wang et al., 2020). Although several novel microbiology biomarkers had been described as having a diagnostic role in lung diseases, it still needs to be further confirmed by larger-scale clinical studies.

In the present study, the included literature usually involved processing of 16S rRNA amplicon sequencing or PCR method to detect the abundance or expression level of microbiome. However, there are several limitations. First, the lung microbiota community was different because of the differences of ethnicity, nationality, and geographical location, which may induce the deviation in different investigations. Second, further replication in large population-based studies is necessary to confirm these results, which should be in the same population (COPD, asthma, or other disease) with the same kind of sample because it is totally different to compare the microbiome from the lungs to that from the upper tract respiratory. Therefore, the correlation between lung microbiology biomarkers and clinical application should be cautiously interpreted. Third, samples in the included studies were different from each one, which may generate inter-study heterogeneity. In the meantime, although subgroup analysis based on ethnicity, age, sample type, method, and disease differences was conducted, COPD and asthma were with only two comparisons, which may also decrease the diagnostic value of lung microbiome markers for these two lung diseases. Last, our analysis only included the bacterial microbiome; however, virome was also a key composition of the lung microbial community which had obtained less attention.

In conclusion, as a type of special marker, microbiota could be a promising indicator for the diagnosis of lung disease. Our results provide essential data to confirm this point. However, it is still difficult to identify the different stages of the disease through the biomarker’s expression values. Therefore, further studies are needed to define the expression values for diagnosis of lung diseases. In the future, more research needs to focus on the diagnosis of lung diseases using novel bacteria biomarkers, which could be used to conduct a more sensitive and accurate biomarker system for the diagnosis of lung diseases.

## Data Availability Statement

The original contributions presented in the study are included in the article/**Supplementary Material**. Further inquiries can be directed to the corresponding authors.

## Author Contributions

RR and CS designed the study, performed the meta-analysis, and wrote the draft. QW performed the literature search. XMD and XYD extracted the data and processed the raw data. XMD also designed the study and wrote the manuscript. XL contributed to the revised manuscript. All authors contributed to the article and approved the submitted manuscript.

## Funding

This work was supported by the Health Bureau of Jiangsu Province (Z2020054) and the Health Bureau of Zhejiang Province (2020KY383).

## Conflict of Interest

The authors declare that the research was conducted in the absence of any commercial or financial relationships that could be construed as a potential conflict of interest.

## Publisher’s Note

All claims expressed in this article are solely those of the authors and do not necessarily represent those of their affiliated organizations, or those of the publisher, the editors and the reviewers. Any product that may be evaluated in this article, or claim that may be made by its manufacturer, is not guaranteed or endorsed by the publisher.
