# Supplementary material for: Microbiota Emergencies in the Diagnosis of Lung Diseases: A Meta-Analysis
Source: Front Cell Infect Microbiol. 2021 Sep 21;11:709634. doi: 10.3389/fcimb.2021.709634 (PMC8490768; doi:10.3389/fcimb.2021.709634)
Supplement: Supplementary file 1 [file DataSheet_1.docx]

Supplementary Material

# Supplementary Figures

#
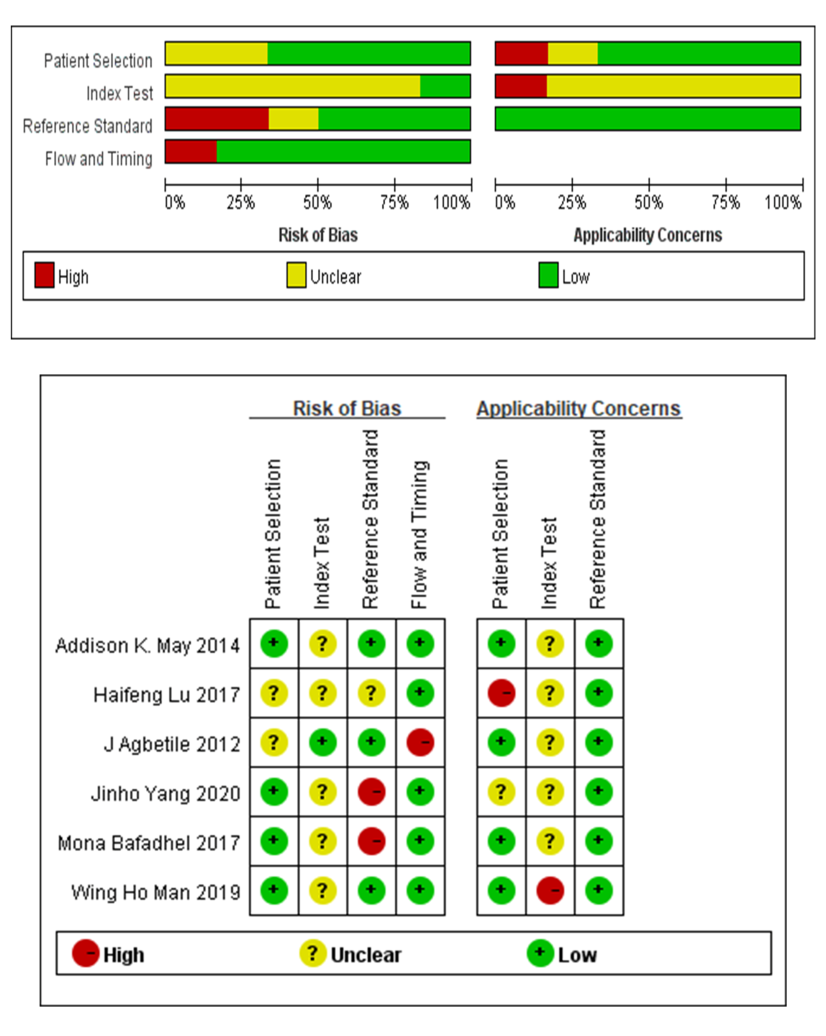


# Supplementary Figure 1. The quality assessment of the included articles by the QUADAS-2 tool.
